# *Drosophila* CG3303 is an essential endoribonuclease linked to TDP-43-mediated neurodegeneration

**DOI:** 10.1038/srep41559

**Published:** 2017-01-31

**Authors:** Pietro Laneve, Lucia Piacentini, Assunta Maria Casale, Davide Capauto, Ubaldo Gioia, Ugo Cappucci, Valerio Di Carlo, Irene Bozzoni, Patrizio Di Micco, Veronica Morea, Carmela Antonia Di Franco, Elisa Caffarelli

**Affiliations:** 1 Center for Life Nano Science@Sapienza, Istituto Italiano di Tecnologia, Viale Regina Elena 291, 00161 Rome, Italy; 2 Department of Biology and Biotechnology, Sapienza University of Rome, P.le A. Moro 5, 00185 Rome, Italy; 3 Institute Pasteur Fondazione Cenci-Bolognetti, Sapienza University of Rome, P.le A. Moro 5, 00185 Rome, Italy; 4 Institute of Molecular Biology and Pathology, National Research Council, Sapienza University of Rome, P.le A. Moro 5, 00185 Rome, Italy; 5 Department of Biochemical Sciences, Sapienza University of Rome, P.le A. Moro 5, 00185 Rome, Italy

## Abstract

Endoribonucleases participate in almost every step of eukaryotic RNA metabolism, acting either as degradative or biosynthetic enzymes. We previously identified the founding member of the Eukaryotic EndoU ribonuclease family, whose components display unique biochemical features and are flexibly involved in important biological processes, such as ribosome biogenesis, tumorigenesis and viral replication. Here we report the discovery of the *CG3303* gene product, which we named DendoU, as a novel family member in *Drosophila*. Functional characterisation revealed that DendoU is essential for *Drosophila* viability and nervous system activity. Pan-neuronal silencing of *dendoU* resulted in fly immature phenotypes, highly reduced lifespan and dramatic motor performance defects. Neuron-subtype selective silencing showed that DendoU is particularly important in cholinergic circuits. At the molecular level, we unveiled that DendoU is a positive regulator of the neurodegeneration-associated protein dTDP-43, whose downregulation recapitulates the ensemble of *dendoU*-dependent phenotypes. This interdisciplinary work, which comprehends *in silico, in vitro* and *in vivo* studies, unveils a relevant role for DendoU in *Drosophila* nervous system physio-pathology and highlights that DendoU-mediated neurotoxicity is, at least in part, contributed by dTDP-43 loss-of-function.

We previously identified and characterised the amphibian XendoU protein^1^,^2^,^3^. XendoU is an endoribonuclease involved in the biogenesis of small nucleolar RNAs, which participate in ribosome function^4^,^5^. Three-dimensional structure determination revealed that XendoU had a novel fold^3^, which led it to be defined the founding member of the Eukaryotic EndoU ribonuclease family by the Structure Classification of Proteins Database (SCOP)^6^. We later characterised another member of the same family, closely related to XendoU, namely human Placental Protein 11 (PP11)^7^, which is highly tissue-specific and represents a tumour-marker in several cancers. Proteins distantly related to XendoU family members are encoded by viruses. They represent genetic markers of nidoviruses^8^, which are responsible for human respiratory diseases, and the Nsp15 prototype is essential in the viral replication cycle^9^,^10^. Eukaryotic and viral endoribonucleases share a unique combination of biochemical features: Mn^2+^-dependent enzymatic activity, uridylates specificity, and release of products with 2′, 3′-cyclic phosphate termini^1^,^7^.

In this study we investigated the role of XendoU homologs in *Drosophila melanogaster*. Sequence comparison detected the two protein products encoded by *CG2145* and *CG3303* genes as XendoU homologs. Deep biochemical analyses revealed that CG3303 product alone shares the aforementioned biochemical features. We named this protein, which is annotated by the major databases as serine protease, DendoU (*Drosophila* endoribonuclease U-specific). By exploiting the powerful reverse-genetics tools of the fruit fly model system, we investigated DendoU *in vivo* activity. The protein was found to be essential for *Drosophila* viability and crucial for neuronal activity. Depletion in the nervous system resulted in severe abnormalities such as immature phenotypes, highly reduced lifespan and impaired locomotion behaviour. These phenotypes overlap with those caused by loss- and gain-of-function of the neurodegeneration-associated protein dTDP-43, which we revealed to be regulated by DendoU.

## Results

### Sequence and structure analyses of *Drosophila* XendoU homologs

Sequence analysis highlighted the occurrence of two *Drosophila* proteins homologous to *X. laevis* XendoU, encoded by *CG2145* and *CG3303* genes (www.flybase.org). The *CG2145* product is assigned an endoribonuclease activity, whereas the *CG3303* product is annotated as putative serine-protease by the UniProt (http://www.uniprot.org/), NCBI (www.ncbi.nlm.nih.gov) and GO (http://gowiki.tamu.edu/wiki/index.php/Main_Page) databases.

**Figure 1:**
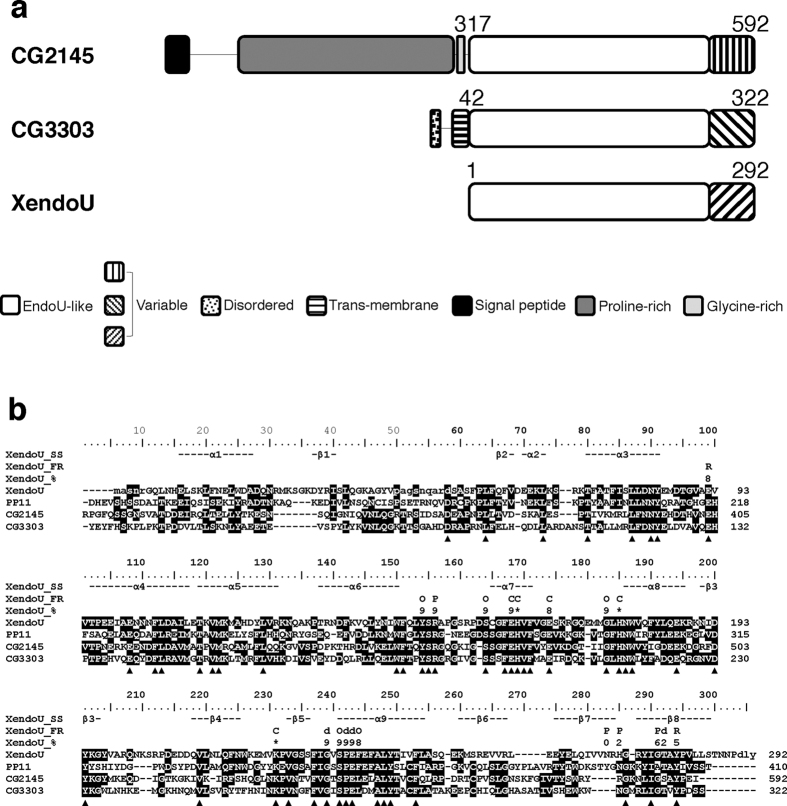
Sequence analyses of *Drosophila* CG2145 and CG3303 gene products. (**a**) Scheme of CG2145 and CG3303 compared to XendoU. Sequence numbering indicates starting and ending residues of XendoU and the CG2145 and CG3303 regions homologous to XendoU (see panel b). These regions comprise a highly conserved segment (EndoU-like, white boxes) and a variable segment (striped boxes). The N-terminal regions of CG2145 and CG3303 comprise additional segments not present in XendoU. The N-terminal region of CG2145 is significantly longer and comprises a signal peptide (black box), a long disordered and proline-rich region (gray box), and a short glycine-rich segment (light gray box). The N-terminal region of CG3303 comprises a disordered segment (dotted box) and a predicted trans-membrane region (horizontally-striped box). (**b**) Multiple sequence alignment of CG3303 and CG2145 products with human PP11 and *X. laevis* XendoU. Residues that are not visible in the experimental XendoU structure are lower-case. Identical residues at the same alignment position have a black background. Positions where all residues are identical are indicated by black triangles. XendoU_SS: secondary structure elements detected in the 3D XendoU structure; α-helices and β-strands are indicated with α and β and a number indicating their progression along the amino acid sequence. XendoU_FR: XendoU residues (i) with a key functional role, i.e., those reported to diminish (**d**) or abolish (C) catalysis if mutated; affect RNA binding (R); in contact with the phosphate molecule in XendoU 3D structure (P); and (ii) shown to interact with XendoU key-residues in the 3D structure (O). XendoU_%: conservation of XendoU residues in a multiple sequence alignment comprising over 700 family members (see Supplementary Table 1); numbers 0, 1, 2, …, 8, 9 indicate conservation in the ranges: <10%, 10–19%, 20–29%, …, 80–89%, 90–99%, respectively; catalytic residues, which are 100% conserved in the extended multiple sequence alignment, are indicated by^∗^.

Comparison of *Drosophila* and XendoU protein sequences showed significant differences in length (592 amino acids for CG2145 and 322 residues for CG3303), which are mostly contributed by the N-terminal regions (Fig. 1a). The C-terminal regions, instead, have similar length (281 and 276 amino acids, respectively) and high sequence identity (41%). The multiple sequence alignment of CG3303 and CG2145 with XendoU and human PP11 homologues, shown in Fig. 1b, highlighted that the 1–250 region of XendoU, comprising most of the residues previously reported to be involved in catalytic activity^2^, was well conserved in both *Drosophila* isoforms and PP11. Conversely, the 251–292 region is more variable in both length and sequence. This region contains several residues, including N270, H272, G277, T278 and Y280, involved in binding the RNA substrate based on the experimentally determined XendoU structure^3^. The high sequence variability in this region indicates that the mode of RNA substrate binding is likely to be different between the two *Drosophila* proteins.

The same conservation pattern shown in Fig. 1b is also observed in a multiple sequence alignment extended to over 700 XendoU homologs (Supplementary Table 1). Here, the three XendoU residues whose point mutation abolished catalysis (i.e., H162, H178, K224) are 100% conserved, most of the other residues whose mutation affects catalysis being conserved in >90% of the sequences (see Fig. 1b and Supplementary Table 1)^2^. Conversely, in the 251–292 variable region, only two of the RNA binding residues are conserved in >50% of the homologous sequences.

These data indicate that the XendoU regions involved either in the catalytic mechanism alone or in both catalysis and RNA binding are more conserved among family members than those responsible for RNA binding only.

### Biochemical characterisation of CG2145 and CG3303 activities

Due to their poor solubility as recombinant enzymes, CG2145 and CG3303 proteins were *in vitro* translated, together with luciferase as control (Supplementary Fig. 1a). To investigate RNase activity, RNA processing assays were performed in the presence of Mn^2+^ as described^1^. Oligoribonucleotides P1 and P2 were used as substrates. P1 contains a natural XendoU cleavage site comprising a 3 U stretch; P2 is a P1 variant where the 3 U stretch is replaced by 2 Us^1^.

**Figure 2:**
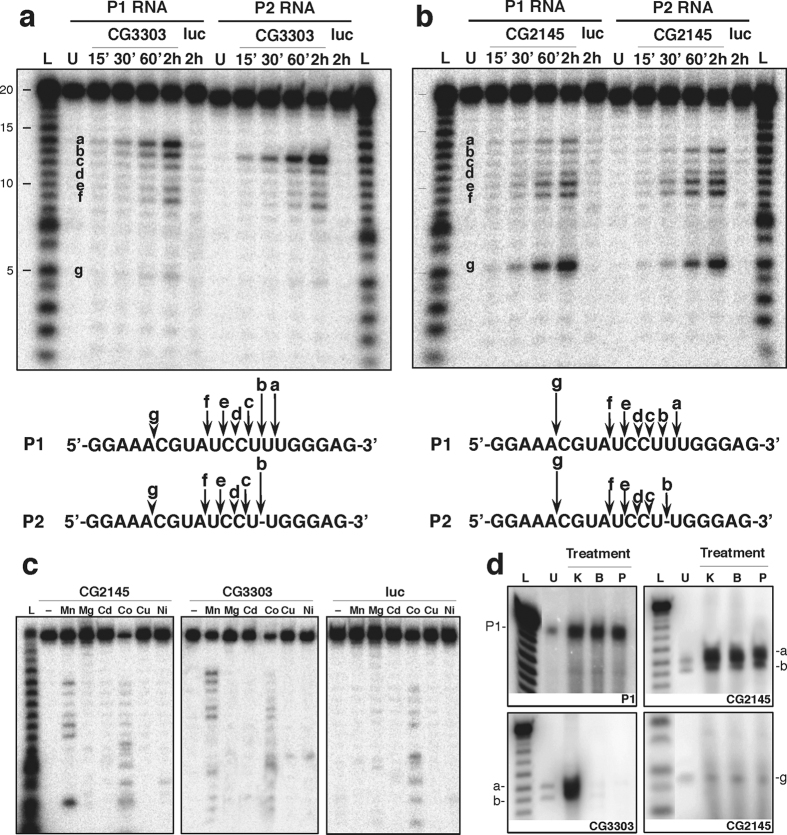
Biochemical analysis of CG3303 and CG2145 proteins. (**a**,**b**) P1 (lanes P1 RNA) or P2 (lanes P2 RNA) oligoribonucleotides were incubated for increasing times (indicated above each lane) with *in vitro* translated CG3303 (**a**) or CG2145 (**b**), or control (luciferase, lanes luc) (see Supplementary Fig. 1a). Unprocessed RNAs were run in lanes U. 1 bp-step ladders, derived from P1 or P2 hydrolysis, are fractionated in lanes L. Letters “a-g” point to cleavage products. Bottom: schemes of P1 and P2 sequences and cleavage site positions. Long arrows, short arrows and arrowheads point to preferential cleavage sites, sites cleaved less efficiently and minor sites, respectively (quantification in Supplementary Fig. 1b,c). (**c**) CG2145 (lanes CG2145) and CG3303 (lanes CG3303) cleavage assays on P1 in the presence of different cations indicated above each lane. Luciferase-expressing lysates (lanes luc) were used in control reactions. RNA ladder is fractionated in lane L. (**d**) Characterization of 3′ end cleavage products released by CG2145 (panels CG2145) or CG3303 (panel CG3303); “a”, “b” and “g” indicate the specific cleavage products analysed. Cleavage product termini were labelled after treatment with alkaline phosphatase (lane P), kinase (lane K) or buffer (lane B). In lanes U, untreated molecules were loaded as markers. Full-length P1 was used as positive control for linear 3′ end (panel P1). RNA ladders were fractionated in lanes L. The full-length versions of the cropped gels are reported in Supplementary Figure 7a.

The time course in Fig. 2a shows the CG3303 RNA cleavage products. As defined by densitometric analysis (Supplementary Fig. 1b) “a” and “b” molecules (long arrows in the scheme) are the major products deriving from cleavages between U residues in P1. Products “c”, “e” and “f” are less abundant (short arrows) deriving from cleavages between CU, UC and AU respectively; “d” and “g” products (arrowheads), are visible only after prolonged incubation. A similar cleavage pattern was observed on P2. Altogether, these results indicate that CG3303 preferentially cleaves at oligo(U).

RNA processing assays with *in vitro* translated CG2145 (Fig. 2b) clearly show that preferential cleavage occurs between A and C nucleotides (“g” products, long arrows in the scheme). The other products derive from less efficient cleavages (Supplementary Fig. 1c).

To analyse ion-dependence of cleavage, P1 processing reactions were performed in the presence of Mn^2+^ or different cations. Figure 2c shows that only Mn^2+^ triggered CG3303 and CG2145 specific activities, whereas Co^2+^ induced unspecific cleavages as in luciferase control (lanes Co). These results indicate that the two *Drosophila* enzymes are Mn^2+^-dependent endoribonucleases.

Finally, the chemistry of the reaction was determined by analysing the 3′ ends of the P1 products generated by CG3303 or CG2145. Unlabelled cleavage products “a”, “b” and “g” (indicated aside each gel of Fig. 2d), and full-length P1 as control, were conjugated with 5′-[^32^P]pCp upon differential enzymatic treatments. Specifically, alkaline phosphatase treatment (lanes P) removes phosphate only from linear ends, whereas kinase treatment removes 2′-3′-cyclic phosphate (lanes K)^1^. As shown in Fig. 2d, the “a” and “b” cleavage products generated by CG3303 are labelled only after kinase treatment, indicating the occurrence of cyclic phosphate termini (panel CG3303); conversely, CG2145 generates products with linear 3′ ends that are labelled upon both treatments (panels CG2145). These results indicate that the two enzymes act through different catalytic mechanisms.

Based on biochemical features, we assigned CG3303 product to the Eukaryotic EndoU ribonuclease family and named it DendoU (*Drosophila* endoribonuclease U-specific).

### *CG2145* and *dendoU* expression profiles and loss-of-function analyses

**Figure 3:**
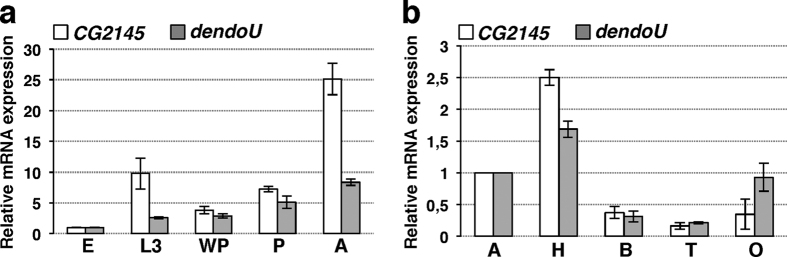
*CG2145* and *dendoU* expression profiles. (**a**) qRT-PCR quantification of *CG2145* (white bars) and *dendoU* (grey bars) mRNAs in embryos (E), larvae (L3), white pre-pupae (WP), pupae (P) and adults (A), relative to *rp49* as control. Expression levels were set as 1 in embryos. (**b**) qRT-PCR quantification of *CG2145* (white bars) and *dendoU* (grey bars) mRNAs in adult flies; whole adults (A), heads (H), bodies (B), testis (T), ovaries (O). Expression levels in whole adult were set as 1.

We analysed temporal expression of *CG2145* and *dendoU* at specific *Drosophila* developmental stages (Fig. 3a). *CG2145* and *dendoU* mRNAs progressively accumulated throughout development, and showed a 25- and 8-fold increase in adults compared to embryos, respectively. Spatial expression profiles in adult somatic and germinal tissues revealed that *CG2145* and *dendoU* mRNAs were highly expressed in *Drosophila*’s head (Fig. 3b).

To delve into *CG2145* and *dendoU* function, we analyzed their mutant phenotypes. For *CG2145*, we exploited the insertional mutant P{EP}CG2145^G605 ^11. It produced 80% lower mRNA levels and undetectable protein levels compared to wild type (Supplementary Fig. 2a,b). Mutant flies were viable and fertile, without noticeable behavioural or developmental phenotypes (Supplementary video 1).

Since no mutant alleles were available for *dendoU*, we exploited the GAL4/UAS binary system^12^ to induce *dendoU* silencing *in vivo* by transgenic double-stranded RNA interference (RNAi)^13^.

Ubiquitous *dendoU* dsRNA expression, driven by *actin-GAL4,* induced *dendoU* mRNA decrease by about 60% in the interfered larvae compared to control (Supplementary Fig. 2c) and resulted in late pupal lethality. This finding indicates that *dendoU* is an essential gene in *Drosophila.* Therefore, we deepened our analyses on DendoU. Unfortunately, reiterated efforts for generating specific antibodies against DendoU protein were not successful, likely due to the shortness of the region diversifying DendoU from CG2145 (see Fig. 1a).

### Neuronal silencing of *dendoU* expression severely impairs fly lifespan, development and motor performance

Since eyes and brain are the major components of *Drosophila’s* head, where *dendoU* is predominantly expressed, we used the developing eye-specific GMR-GAL4, or the pan-neuronal elav-GAL4 drivers for *dendoU* tissue-specific silencing. GMR-GAL4 mediated *dendoU* RNAi *in vivo* did not produce any significant eye phenotype (Supplementary Fig. 3), suggesting that the gene is not directly involved in ommatidia proliferation/differentiation.

**Figure 4:**
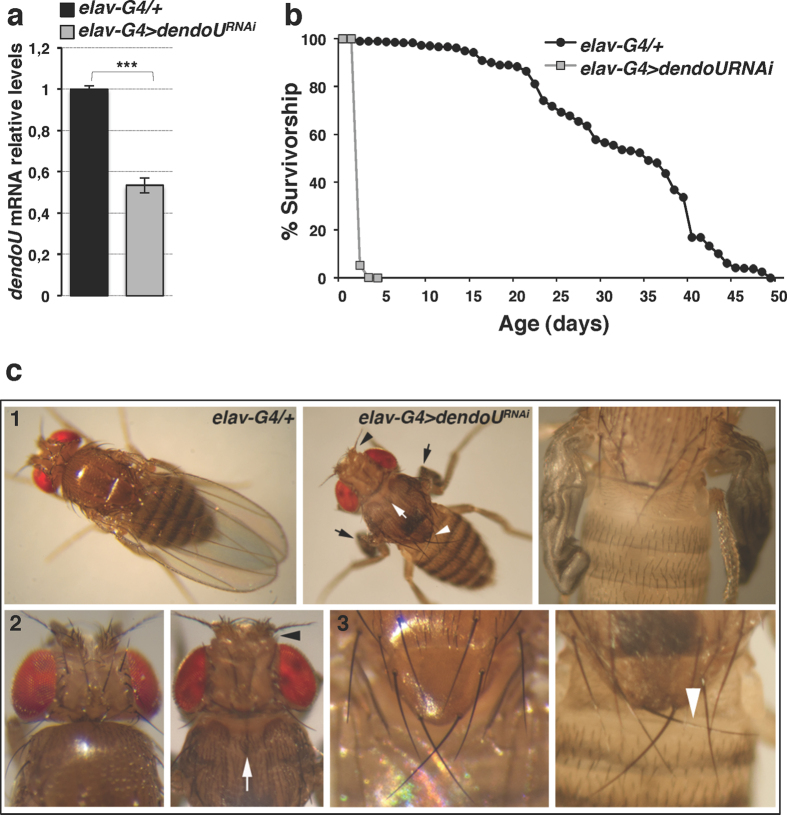
Functional effects of *dendoU* inactivation in nervous system. (**a**) *dendoU* mRNA qRT-PCR quantification in pan-neuronal interfered *Drosophila* heads (grey bar). *Rp49* served as a standard. Expression levels in control flies (black bar) were set as 1. (**b**) Quantification of fly median lifespan. Average values were 32.07 ± 0.43d and 2.05 ± 0.01d in control (black circles) and pan-neuronal *dendoU* silenced flies (grey squares), respectively. The log rank test showed that the two curves were significantly different (P < 0.001). (**c**) Compared to control flies (panels 1, 2 and 3), pan-neuronal *dendoU* depleted flies showed unexpanded wings (black arrows), dimpled thorax (white arrow), unretracted ptilinum (headed black arrow), and misoriented scutellar bristles (headed white arrow).

Importantly, *dendoU* silencing in fly nervous system through elav-GAL4 driver (Fig. 4a) caused dramatic effects (Fig. 4b,c and Supplementary Table 2). Besides pupal lethality (17%) and eclosion defects (3%), a drastic reduction in median lifespan was observed (the median lifespan was 32.07 ± 0.43 days for control flies *vs* 2.05 ± 0.01 days for *elav-G4* > *dendoU*^RNAi^ flies) (Fig. 4b). Moreover, adult flies displayed several abnormalities (Fig. 4c). They appeared as immature animals bearing unexpanded wings, soft cuticle, unretracted ptilinum, dimpled dorsal thorax, and misoriented scutellar bristles. Remarkably, immature-looking flies also showed severe locomotor defects and marked uncoordination. Compared to control flies (Supplementary video 2), pan-neuronal interfered flies were quite unable to walk or stay upright, and usually became stuck in the culture medium immediately after eclosion. Impaired body posture and overall weakness were so severe that standard climbing assay could not be performed. Therefore, locomotor activity of these flies was assessed by evaluating voluntary locomotion on a horizontal surface (Supplementary video 3). As shown in the video, walking assays revealed substantial impairment of motor capacities. Flies were unable to move and promptly restore themselves to an upright position after being knocked down to the bottom of a dish by gentle agitation. The same immature phenotype and locomotor defects were observed in an independent RNAi line, upon exclusion of any off-target effect (Supplementary Fig. 4a,b and Supplementary video 4).

### DendoU activity is required in motor and cholinergic neurons

Once established that *dendoU* silencing in neuronal cells impaired both the last step of metamorphosis and locomotion, we investigated which neuronal subset requires this activity.

Since pupal-to-adult transition is coordinated by the CCAP/bursicon neuronal network^14^, we first silenced *dendoU* expression in CCAP/bursicon neurons. Adult flies were viable and did not show any morphological defect (Supplementary Fig. 5a and Supplementary Table 2). This suggests that the immature phenotype caused by pan-neuronal *dendoU* knockdown is not cell-autonomous. Accordingly, analysis on late pupal heads and thoracic-abdominal ganglia, where CCAP/bursicon neuron subset resides^15^, revealed that the expression of transcripts relevant for their neuronal activity (i.e., *bursicon* hormone, its partner *p-bursicon* and their receptor *dlgr2*) was unaffected in *dendoU* interfered flies (Supplementary Fig. 5b).

**Figure 5:**
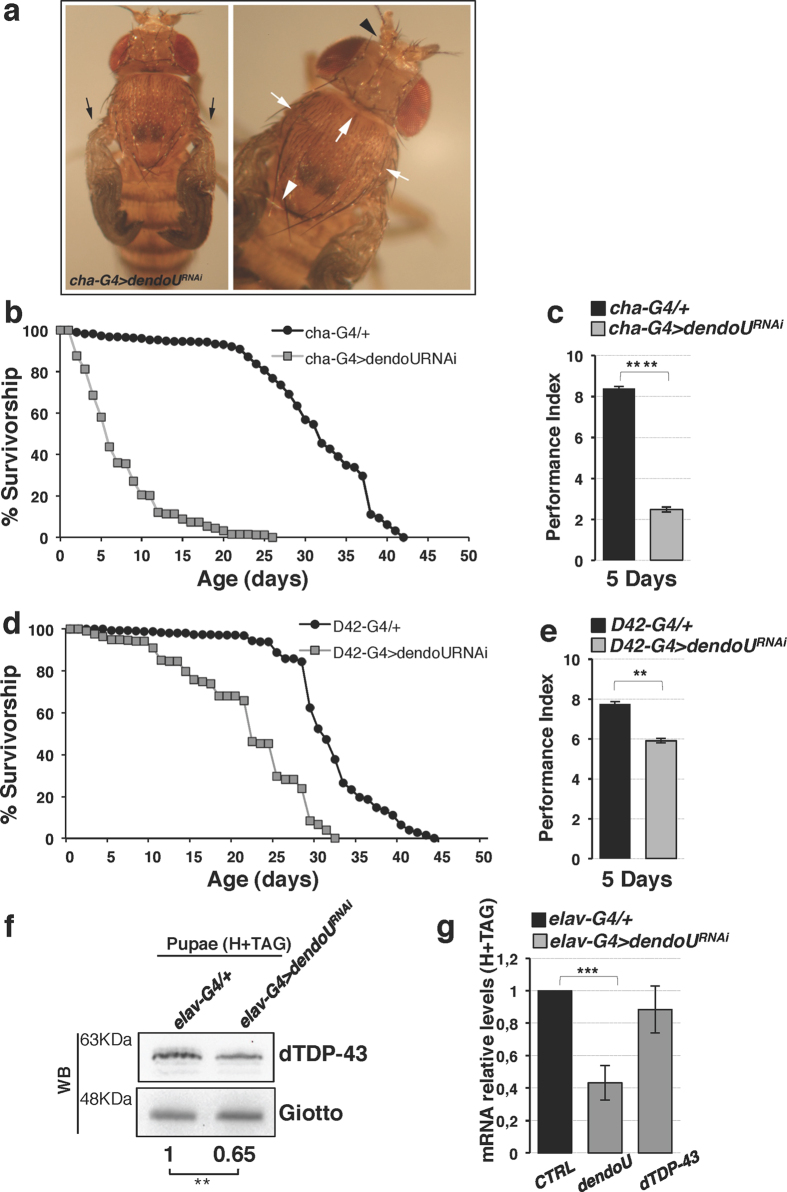
Functional effects of *dendoU* inactivation in neuron subtypes. (**a**) Juvenile phenotype in *cha-G4* > *dendoU*^*RNAi*^ flies. Morphological defects are indicated as in Fig. 4c. (**b**) Diagram showing viability in *cha-G4* > *dendoU*^*RNAi*^ flies. Median lifespan in experimental flies (grey squares) was 7.57 ± 0.18d, whereas it was 31.06 ± 0.37d in controls (black circles). The log rank test showed that the two curves were significantly different (P < 0,001). (**c**) Flies with indicated genotypes and age were tested using a standard climbing assay. The performance index (expressed as the percentage of flies that climbed to the top vial relative to the total number of tested flies) was significantly lower in *cha-G4* > *dendoU*^*RNAi*^ flies (grey bar) compared to controls (black bar). All average data are presented as mean ± SEM (*n* ≥ 100 flies per genotype) and compared with 2-tailed unpaired *t*-tests. Tests were performed using Prism (GraphPad Software, Inc.). *P* value, ****p < 0.0001. (**d**) Diagram showing viability in *D42-G4 > dendoU*^*RNAi*^ flies. Median lifespan in experimental flies (grey squares) was 21.53 ± 0.50d and it was 31.38 ± 0.26d in control flies (black circles). The log rank test showed that the two curves were significantly different (P < 0.001). (**e**) Locomotor deficit in 5-day*-*old flies interfered for *dendoU* in motor neurons. The climbing performance in *D42-G4* > *dendoU*^*RNAi*^ flies (grey bar) was significantly lower compared to control flies (black bar). Statistics as in panel c. *P* values, **p = 0.0015. (**f**) Western blot of dTDP-43 in head and thoracic-abdominal ganglia (H + TAG) dissected from pan-neuronal *dendoU* interfered pupae. Giotto was used as internal control. Result was expressed as means for at least three independent biological replicates. (**g**) qRT-PCR analysis of *dendoU* and *dTDP-43* mRNAs (grey bars) in the same samples as in (f). Expression levels in control samples are set as 1 (black bar). Data are presented as mean ± SD in triplicates (***P < 0.001, Paired T-test). The full-length versions of the cropped blots are shown in Supplementary Figure 7b.

To investigate whether the immature phenotype could be ascribed to an impaired synaptic input to CCAP/bursicon neurons, we silenced *dendoU* expression in cholinergic neurons^16^. We used choline acetyltransferase (Cha) promoter-driven GAL4, which is expressed in both central and sensory cholinergic neurons. *DendoU* downregulation resulted in the same immature phenotypes observed in pan-neuronal knockdown (Fig. 5a and Supplementary Table 2). This result indicates that *dendoU*-linked immature phenotype can be, at least partially, ascribed to cholinergic DendoU function. *DendoU* silencing in cholinergic neurons also produces relevant effects on median lifespan, with 75% decrease (31.06 ± 0.37 days for control flies *vs* 7.57 ± 0.18 days for cha-G4 > *dendoU*^RNAi^) (Fig. 5b and Supplementary Table 2), and locomotion, with 70% reduction (Fig. 5c and Supplementary Table 2). To verify whether DendoU effect on locomotion is cell-autonomous, functional inactivation of *dendoU* in motor neurons was performed by the specific D42-GAL4 driver. Climbing assays showed 25% decrease of performance index (Fig. 5d and Supplementary Table 2), indicating the involvement of DendoU in motor neuron activity. Moreover, *dendoU* silencing in motor neurons caused a low frequency of altered phenotypes, i.e. 3% of immature phenotype and 7% of mild immature phenotypes (unexpanded wings), and 30% decrease of median lifespan (31.38 ± 0.26 days for control flies *vs* 21.53 ± 0.50 days for D42-G4 > dendoU^RNAi^ flies) (Fig. 5e and Supplementary Table 2).

### DendoU controls dTDP-43 protein levels

The ensemble of phenotypes that we observed upon *dendoU* pan-neuronal knockdown, although more severe, largely overlaps with that linked to dTDP-43 deregulation in fly nervous system (compare Supplementary videos 3 and 5)^17^. In particular, both loss and gain of dTDP-43 caused reduced lifespan^18^, immature phenotypes^19^, locomotion defects^18^ and impaired synaptic transmission, which represents one of the earliest events in neuronal degeneration^20^.

Indeed, *dendoU* pan-neuronal silencing resulted in 35% decrease of dTDP-43 protein in late pupal heads and thoracic-abdominal ganglia, compared to control (Fig. 5f). However, *dTDP-43* mRNA levels showed no alteration (Fig. 5g). These results were confirmed by the analysis of the second *dendoU* RNAi line, which showed a comparable decrease of dTDP-43 protein without any alteration of mRNA level (Supplementary Fig. 6a,b).

These results indicate that DendoU, through a still unidentified mechanism, controls dTDP-43 protein levels.

## Discussion

Based on sequence similarity and domain organization, both CG2145 and DendoU endoribonucleases should be assigned to the Eukaryotic EndoU ribonuclease family. However, deep biochemical characterization revealed distinct features in sequence-specificity and cleavage chemistry. This suggests that they recognize and/or cleave different RNA substrates and act through different catalytic mechanisms *in vivo*. On this basis, we propose to include only DendoU, which was annotated in databases as putative serine-protease, in the aforementioned family. These findings underline the relevance of combining biochemical and sequence/structure analyses to elucidate protein function.

Taking advantage of *Drosophila in vivo* model system to investigate the contribution of target genes to biological processes, we demonstrated that DendoU is essential for fly viability whereas CG2145 is not. Indeed, DendoU systemic silencing resulted in pupal lethality, indicating that *in vivo* activity of DendoU is not redundant.

We unveiled a crucial role for DendoU in *Drosophila* nervous system physiology and pathology. Remarkably, some of the phenotypes determined by *dendoU* pan-neuronal silencing, i.e., pupal lethality, strongly reduced fly lifespan, juvenile phenotypes and severely impaired locomotion, overlap with those caused by dTDP-43 deregulation^17^. TDP-43 is an evolutionary conserved RNA/DNA binding protein, considered to be a neuronal activity-responsive factor and neurodegeneration hallmark^21^,^22^. As shown in different model systems, TDP-43 cellular levels must be exquisitely regulated since even subtle alterations result in neurotoxicity^23^,^24^. We demonstrate, through two independent RNAi lines, that *dendoU* knockdown causes an about 35% decrease of dTDP-43 protein, indicating that one relevant function of DendoU in *Drosophila* nervous system is to contribute to the control of dTDP-43 levels. These data suggest that DendoU-linked phenotypes are at least in part mediated by dTDP-43 loss-of-function. The involvement of other genes is expected in light of the more severe phenotype observed in dendoU^RNAi^ flies compared to dTDP-43^RNAi^. Moreover, since *dTDP-43* mRNA is not a direct DendoU substrate, dTDP-43 regulation must be mediated by still unknown DendoU targets. Ongoing transcriptomics analysis aims at identifying DendoU target genes responsible for the regulation of i) dTDP-43 dependent circuit; ii) other molecular pathways underlying the observed dramatic phenotypes.

Inside the nervous system, DendoU contribution is not restricted to individual neuronal sub-types but extended to complex neuronal networks, largely dependent on cholinergic neurons. Indeed, *dendoU* depletion in this neuronal subclass recapitulates the pan-neuronal phenotypes indicating a defect in excitatory inputs to CCAP/bursicon and motor neurons. This is in line with the already demonstrated defects in synaptic efficacy caused by deregulation of dTDP-43^20^.

In conclusion, we identified a novel member of the Eukaryotic EndoU ribonuclease family with a pivotal role in *Drosophila* nervous system. DendoU functions once more demonstrate the high versatility of this family of enzymes that intervene in cellular pathways as relevant as diverse.

## Methods

### Sequence and structure analyses

The sequences of *D. melanogaster* proteins CG2145 and CG3303 were downloaded from the UniProt Knowledgebase (UniProt IDs: Q9VF14_DROME and ENDOU_DROME, respectively)^25^. Searches for homologues were performed using the program PSI-BLAST^26^ (up to five iterations) at the NCBI (http://blast.ncbi.nlm.nih.gov/).

A multiple sequence alignment comprising all the matched sequences was produced using a local version of ClustalW^27^ (http://www.clustal.org/). This alignment was edited using the Bioedit 7.2.5 package (http://www.mbio.ncsu.edu/bioedit/bioedit.html) to retain only sequences where the three XendoU residues whose point mutation abolished catalysis (i.e., H162, H178, K224) are present. The alignment shown in Supplementary Table 1 was edited by hand to minimize the number of deletions occurring within secondary structure elements.

Predictions of protein features such as occurrence of signal peptides, disordered segments, trans-membrane regions, and secondary structure elements were obtained from several available servers including: Pfam^28^; SMART^29^; PSIPRED^30^; TMHMM^31^; SignalP^32^ and GeneSilico MetaDisorder Service^33^.

### *In vitro* translation of CG2145 and CG3303

Total RNA was purified from whole larvae and adults by tissue homogenization and Qiazol reagent extraction. CG2145 and CG3303 ORFs were amplified, through the oligonucleotides A (5′-ATGCGCTGCCTGGCATTGAGC-3′), B (5′-TCAAATCTCCGGATAGGCGCTGCC-3′) and C (5′-ATGGACGCAGTGCAGCAGCAAAC-3′), D (5′-CTAGGAGCTGTCCGGATAGAC-3′), respectively, and cloned into the pGEX-4T1 vector, between the EcoRI and NotI sites. CG2145 and CG3303 [^35^S]methionine-labeled proteins were produced by *in vitro* transcription and translation reactions using the TNT-coupled Reticulocyte Lysate System kit (Promega), according to manifacturer’s instructions.

### *In vitro* processing assay

Oligoribonucleotides P1 (5′-GGAAACGUAUCCUUUGGGAG-3′) or P2 (5′-GGAAACGUAUCCUUGGGAGG-3′) were 5′-end labeled with [γ-32P]ATP (PerkinElmer Life Sciences) by T4 polynucleotide kinase (New England Biolabs) treatment. Processing reactions were carried out in the conditions already described^1^, by incubating 3 × 10^4^ cpm of ^32^P-labeled substrate together with 5–10 μl of CG2145- or CG3303-expressing reticulocyte lysates, in the presence of 5 mM MnCl_2_ (or other cations, for ion dependence determination experiments). Cleavage products were phenol-extracted and analysed on 20% polyacrylamide, 7 M urea gels. The RNA ladder derived from incubation of P1 oligo (200,000 cpm) in 500 mM NaHCO_3_ at 90 °C for 20 min. Densitometric analysis was performed by Typhoon Trio Imager (Amersham Bioscience), using the ImageQuant 5.0 Software. For each cleavage assay, band intensities were expressed as percentages, relative to the major cleavage product set as 100.

### Analysis of cleavage product 3′-ends

Specific reaction products obtained by incubation of unlabeled P1 oligonucleotide with CG2145- or CG3303-expressing reticulocyte lysates were gel-purified after acrylamide fractionation, phenol-purified and redissolved in 30 mM Tris, pH 8.0, 15 mM MgCl_2_. The mixture was incubated for 45 min at 37 °C in the presence of 1.5 units/μl of T4 polynucleotide kinase to remove the 2′-3′-cyclic phosphate^34^. RNA was treated with kinase buffer or with alkaline phosphatase (Roche Applied Science) as negative controls. Extracted RNA was then labeled by T4 RNA ligase (New England Biolabs) for 5 h at 16 °C, in the presence of 5′-[32 P] cytidine 3′,5′-bisphosphate (Perkin Elmer Life Science) and analyzed on 20% polyacrylamide, 7 M urea gel.

### Drosophila strains

The Ore-R stock used here has been kept in our laboratory for many years under standard conditions. Stocks obtained by Bloomington Stock Center: P{GawB}elav^C155^ (#458); y^1^ w^*^; P{Act5C-GAL4}17bFO1/TM6B, Tb^1^ (#3954); w^*^; P{GawB}D42 (#8816); w^1118^; P{Burs-GAL4.TH}4 M (#51980); w^*^; P{Burs-GAL4.P}P12 (#40972); y^1^ w^*^; Bl^1^/CyO, y^+^; P{CCAP-GAL4.P}9 (#25686); y^1^ w^*^; P{CCAP-GAL4.P}16 (#25685); Cha-GAL4^35^; w^*^; P{GAL4-ninaE.GMR}12 (#1104) and w^*^ P{EP}CG2145^G605^ (#33457). RNAi lines, w^1118^; P{GD3933}v9916 (v9916), P{KK115483}VIE-260B (v109080) and P{KK108354}VIE260B (v104401) were obtained from Vienna Stock Center. *In vivo* RNAi experiments were performed at 25 °C and 29 °C.

### Expression profile and western blot analyses

mRNA and protein levels were determined by quantitative real-time PCR (qRT-PCR) and semi-quantitative PCR analysis (RT-PCR) or by Western blot assay, respectively. For qRT-PCR, RNA extracted from specific samples (developmental stages or tissues) by Qiazol reagent (Qiagen) was used to generate cDNA using SuperScriptTM III First-Strand Synthesis SuperMix (Invitrogen). The qRT-PCR was performed using QuantiFast SYBR Green PCR Kit (Qiagen) or miScript SYBR-Green PCR Kit (Qiagen) through a 7500 Fast Real-Time PCR (Applied Biosystem), using the oligonucletides listed below. The reaction mixtures were kept at 95 °C for 15 min, followed by 40 cycles at 95 °C for 15 s and 60 °C for 1 min. Fluorescence output results were captured and analyzed using 7300 System SDS v1.4 Software (Applied Biosystems), and the threshold cycle (Ct) was used for assessing relative levels of target transcripts versus *rp49* transcripts. For RT-PCR, amplicons were fractionated along a 2% agarose gel and revealed by ChemiDoc XRS + Molecular Imager (Bio-Rad). *Gapdh* mRNA was used as loading control. ImageJ was used for intensity band quantification. For protein level determination, Western blots were carried out using custom antibodies against CG2145 (PRIMM) (1:1000) and dTDP-43^18^ (1:1000), or antibodies against Giotto (provided by G. Cestra) (1:5000).

### qRT- and RT-PCR oligonucleotides

CG2145 F: CGGTAACATCCAAGTGAATCT

CG2145 R: GCTTTCGAGTCCACAGTCAAA

GC3303 F: CTAACTCCACCACCGCTCTG

GC3303 R: ATATTGCTCCTGGACGTGCT

dlgr2 F: TCCAGGAGGAAGAGGACTCA

dlgr2 R: AGGAAGTGGCATGCCTAGAA

pburs F: GCATGTCCAGGAACTGCTCT

pburs R: TCGTCGAACTCCTCCTTGAT

burs F: CAAGAAGGTCCTGACCAAGG

burs R: GCCGGAAGTGATTGTTGAGT

dTDP-43 F: CCTTCAGGGCCTTTAGCTTT

dTDP-43 R: CGAAACGCCCTTGATAATGT

rp49 F: GCGCACCAAGCACTTCATC

rp49 R: TTGGGCTTGCGCCATT

msn F: CAGCAACAACAACTGGCTCC

msn R: GCCCACAACTTCGATGAGCT

### Walking, climbing and survival assays

The assays were performed according to earlier established protocol^18^. For the walking assay, we examined the behaviour of freely walking upright adult flies on a flat horizontal surface. Newly eclosed female and males were transferred in batches of 15 to fresh vials and aged for 3–4 days. The flies were transferred, without anaesthesia, to the centre of 200 mm Petri dish to allow for video recordings using a Nikon D5000 mounted on a WILD M3B (Wild Heerbrugg) stereomicroscope. For the climbing assay we used an apparatus consisting of two empty polystyrene vials vertically joined by tape facing each other. For the lower vial, a vertical distance of 8 cm above the bottom surface was measured and marked by drawing a circle around the entire circumference of the vial. For the climbing assay, a group of 10 male flies were transferred into the lower vial and allowed to acclimatize to the new setting for 1 minute. The climbing assay involved gently tapping the flies down to the bottom of the vial and measuring the number of flies per group that can climb above the 8 cm mark by 15 seconds after the tap, recorded as the percentage success rate. Nine trials were performed for each group and n ≥ 100 flies were assayed for each genotype. Experiments were performed during daylight to minimize potential effects of circadian oscillation. All average data are presented as mean ± SEM and compared with 2-tailed unpaired t-tests. Statistical tests were performed using Prism (GraphPad Software, Inc.). For survival assay, homozygous virgins bearing GAL4 were crossed to homozygous UAS-dendoU^RNAi^ transgene and control Ore-R males. The progeny from these crosses were maintained on standard cornmeal-sucrose-yeast-agar diet at 29 °C. Every 2–3 days, flies were passed into new vials and dead flies were counted. The survival rate was calculated by the percentage of total flies surviving. Survival curves were analysed by the log-rank method using GraphPad Prism (n ≥ 500 flies for all genotypes except for *D42-G4* > *dendoU*^*RNAi*^ (n ≥ 200)).

### Statistics

Statistical significance was determined by two-tailed Student’s t-tests. A p value < 0.05 was considered statistically significant. Data shown here are the mean ± SEM from at least three biological replicates, unless otherwise indicated. All statistics were performed using GraphPad Prism version 6.00 for Windows, GraphPad Software, La Jolla California USA, www.graphpad.com. Lifespan data were analyzed by log rank test and data from qPCR by Mann-Whitney-U Test.

## Additional Information

**How to cite this article:** Laneve, P. *et al. Drosophila* CG3303 is an essential endoribonuclease linked to TDP-43-mediated neurodegeneration. *Sci. Rep.* **7**, 41559; doi: 10.1038/srep41559 (2017).

**Publisher's note:** Springer Nature remains neutral with regard to jurisdictional claims in published maps and institutional affiliations.

## Supplementary Material

Supplementary Information

Supplementary Video 1

Supplementary Video 2

Supplementary Video 3

Supplementary Video 4

Supplementary Video 5

Supplementary Dataset 1
