# Supplementary material for: Drosophila CG3303 is an essential endoribonuclease linked to TDP-43-mediated neurodegeneration
Source: Sci Rep. 2017 Jan 31;7:41559. doi: 10.1038/srep41559 (PMC5282483; doi:10.1038/srep41559)
Supplement: Supplementary Information [file srep41559-s1.pdf]

Drosophila CG3303 is an essential endoribonuclease linked to TDP-43-mediated neurodegeneration

Pietro Laneve[1†], Lucia Piacentini[2,3†], Assunta Maria Casale[2,3], Davide Capauto[1,2], Ubaldo Gioia[2,a], Ugo Cappucci[2], Valerio Di Carlo[2,b], Irene Bozzoni[1,2,3,4], Patrizio Di Micco[5,c], Veronica Morea[4], Carmela Antonia Di Franco[2*], Elisa Caffarelli[1,4*]

[1]Center for Life Nano Science@Sapienza, Istituto Italiano di Tecnologia, Viale Regina Elena 291, 00161 Rome-Italy.
[2]Department of Biology and Biotechnology, Sapienza University of Rome, P.le A. Moro 5, 00185 Rome-Italy.
[3]Institute Pasteur Fondazione Cenci-Bolognetti, Sapienza University of Rome, P.le A. Moro 5, 00185 Rome-Italy.
[4]Institute of Molecular Biology and Pathology, National Research Council, Sapienza University of Rome, P.le A. Moro 5, 00185 Rome-Italy.
[5]Department of Biochemical Sciences, Sapienza University of Rome, P.le A. Moro 5, 00185 Rome-Italy.

† These authors equally contributed to this work
*Corresponding authors

SUPPLEMENTARY FIGURE LEGENDS

Supplementary Figure 1. *In vitro* translation of CG3303 and CG2145 proteins and quantification of their processing products.
Related to Figure 2.
(**a**) SDS-PAGE analysis of *in vitro* translated specific ORFs (lanes CG2145 or CG3303) and of the control (luciferase, lane luc). Molecular weights of [^{35}S]methionine-labelled products are indicated aside.
(**b**) CG3303 and (**c**) CG2145 processing reaction products (indicated in Figure 1a and b, respectively) were quantified through densitometric analysis. Value intensity is relative to major bands (deriving from cleavage at "a" site for CG3303, and "g" site for CG2145), set as 100.

Supplementary Figure 2. Expression of *CG2145* in *CG2145* mutant flies and of *dendoU* in *dendoU*- silenced larvae.
Related to Figures 3 and 4.
(**a**) RT-PCR quantification of *CG2145* mRNA in wild type (lane WT) or *CG2145* mutant (lane CG2145^{G605}) heads. Specific band intensity was quantified relative to *rp49* as internal control, and reported below each lane.
(**b**) Western Blot analysis of CG2145 protein performed in the same conditions as in (**a**). Giotto was used as internal standard.
(**c**) Whole larvae qRT-PCR analysis of *dendoU* mRNA expression upon Actin-GAL4-driven silencing (grey bar), respect to control flies (black bar), set as 1. *Rp49* was used as internal control.

Supplementary Figure 3. DendoU depletion in the adult retina.
Related to Figure 4.
Eyes expressing *GMR-G4>dendoU^{RNAi}* displayed normal sized and regularly arranged ommatidia and did not show any sign of age-dependent neurodegeneration, respect to control flies .

Supplementary Figure 4. Phenotypic effects of pan-neuronal *dendoU* inactivation using an indipendent transgenic *dendoU*^{RNAi} line (P{KK115483}VIE-260B).
Related to Figure 4.
(**a**) The morphological defects recapitulate those observed with *dendoU*^{RNAi} line GD3933: unexpanded wings (black arrows), dimpled thorax (white arrow), unretracted ptilinum (headed black arrow) and misoriented scutellar bristles (headed white arrow).
(**b**) qRT-PCR analysis of *dendoU* and of the potential off-target *msn* in pan-neuronal interfered *Drosophila* heads and thoracic abdominal ganglion (H+TAG).

Supplementary Figure 5. DendoU depletion in CCAP/bursicon neurons.
Related to Figure 4.
(**a**) CCAP/bursicon *dendoU* depleted flies exhibited no morphological defects.
(**b**) qRT-PCR analysis of relevant mRNAs for CCAP/bursicon activity (grey bars) in pupal head and thoracic abdominal ganglion (H+TAG), upon pan-neuronal *dendoU* silencing. Expression levels are set as 1 in control flies (black bar).

Supplementary Figure 6. dTDP-43 protein and mRNA levels in the transgenic *dendoU*^{RNAi} line P{KK115483}VIE-260B.
Related to Figure 5.
(**a**) Western blot of dTDP-43 in head and thoracic abdominal ganglion (H+TAG) dissected from pan-neuronal *dendoU* interfered pupae. Giotto was used as internal control.
(**b**) qRT-PCR analysis of dTDP-43 mRNA in the same samples as in (**a**). Data are presented as mean ± SD in biological triplicates (***P < 0.001, Paired T-test).

Supplementary Figure 7. Full-length versions of cropped gels/blots displayed in main Figures.
Related to Figure 2 and 5.
(**a**) full-length version of gels reported in Figure 2d.
(**b**) full-length version of blots reported in Figure 5f.

Supplementary Figure 1

a

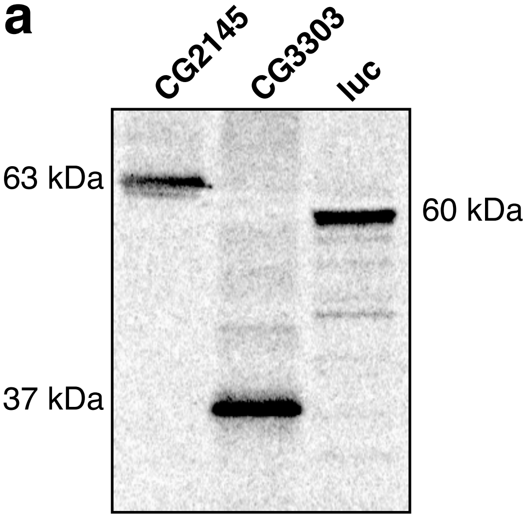

b

CG3303	Substrate P1		Substrate P2	
	**Site**	**Value**	**Site**	**Value**
	a	100,0		
	b	78,9	b	100,0
	c	45,4	c	50,9
	d	24,0	d	25,2
	e	41,0	e	31,1
	f	46,0	f	37,0
	g	20,8	g	7,6

c

CG2145	Substrate P1		Substrate P2	
	**Site**	**Value**	**Site**	**Value**
	a	33,7		
	b	21,7	b	48,1
	c	16,5	c	27,6
	d	34,6	d	48,8
	e	55,4	e	70,1
	f	46,3	f	64,7
	g	100,0	g	100,0

Supplementary Figure 2

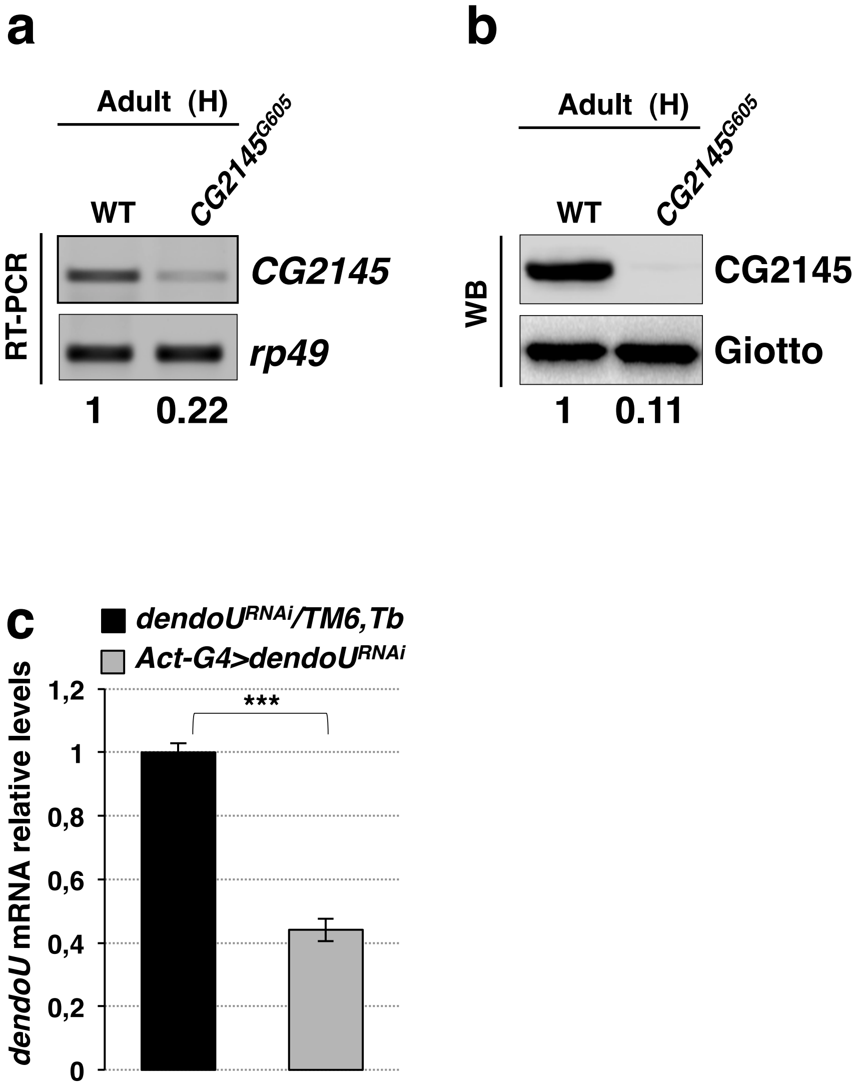

Supplementary Figure 3

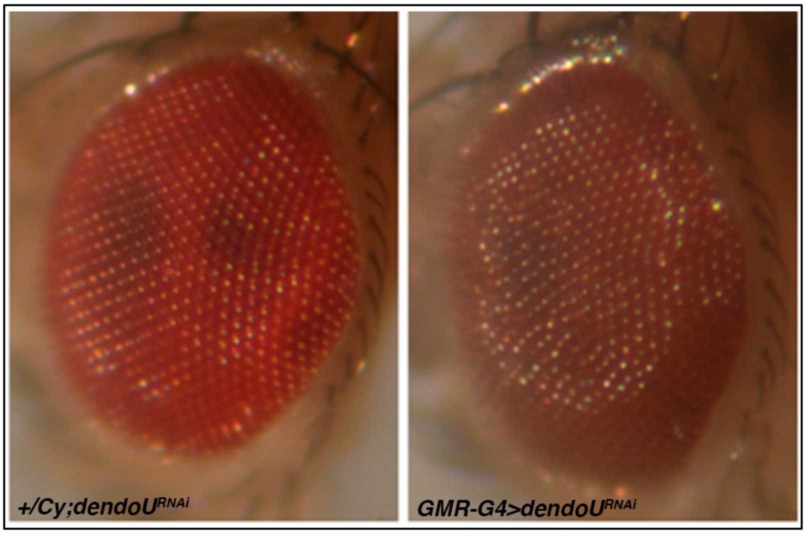

Supplementary Figure 4

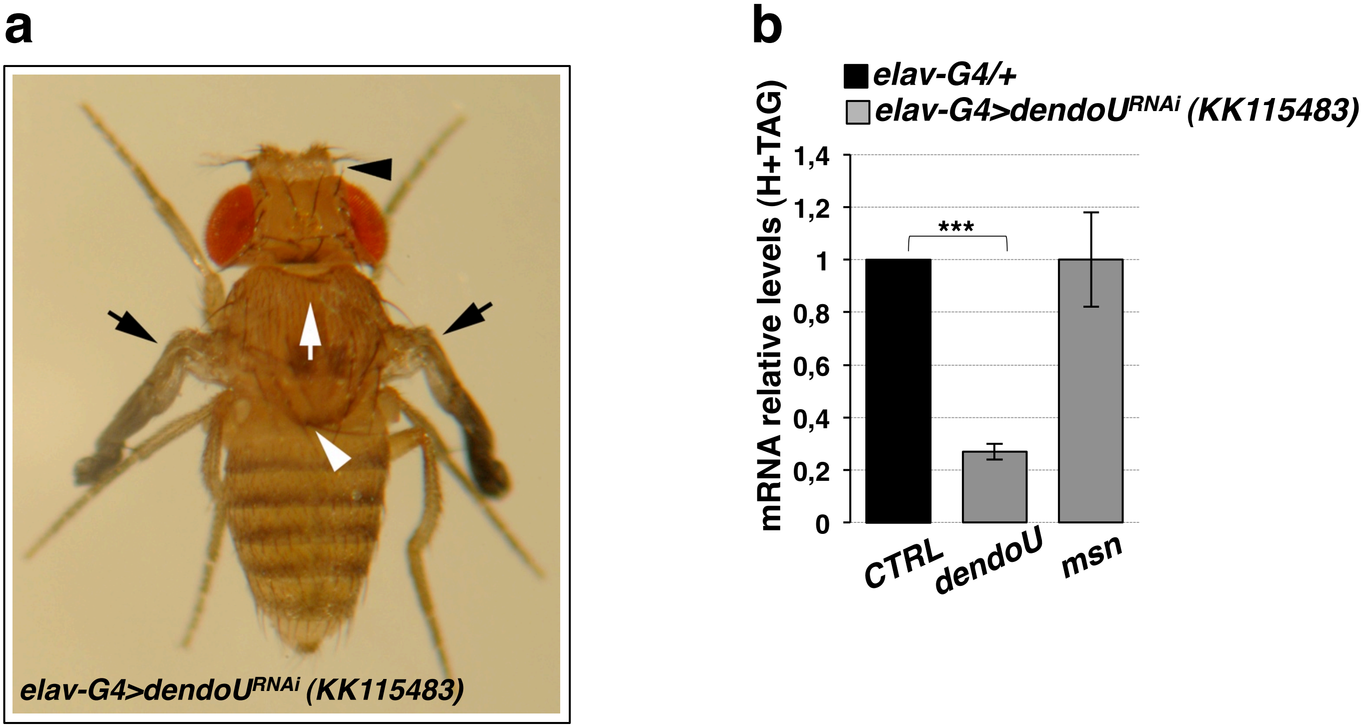

Supplementary Figure 5

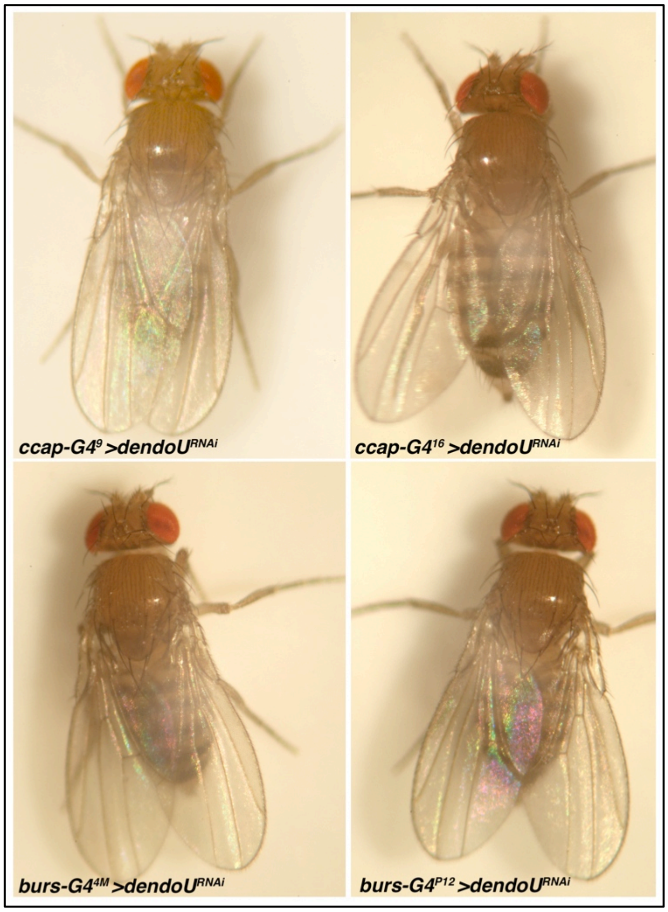

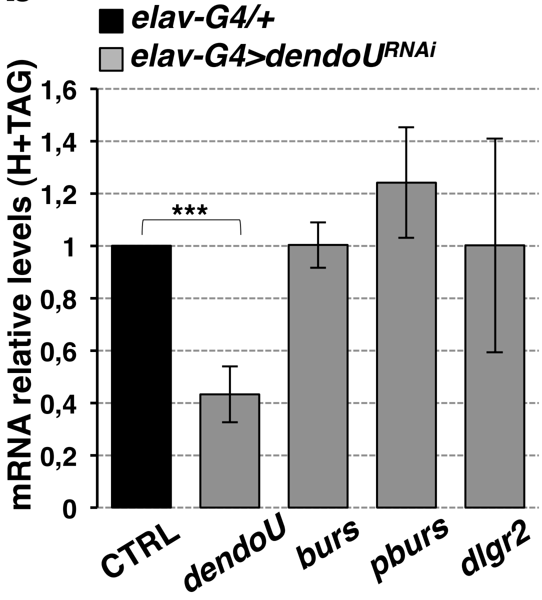

Supplementary Figure 6

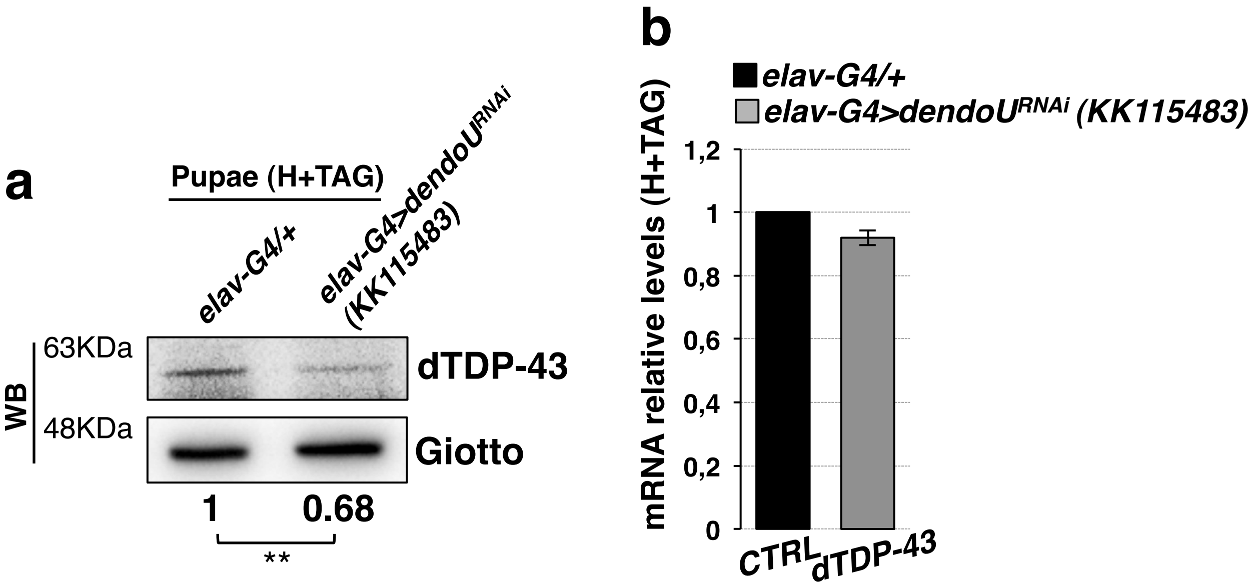

Supplementary Figure 7

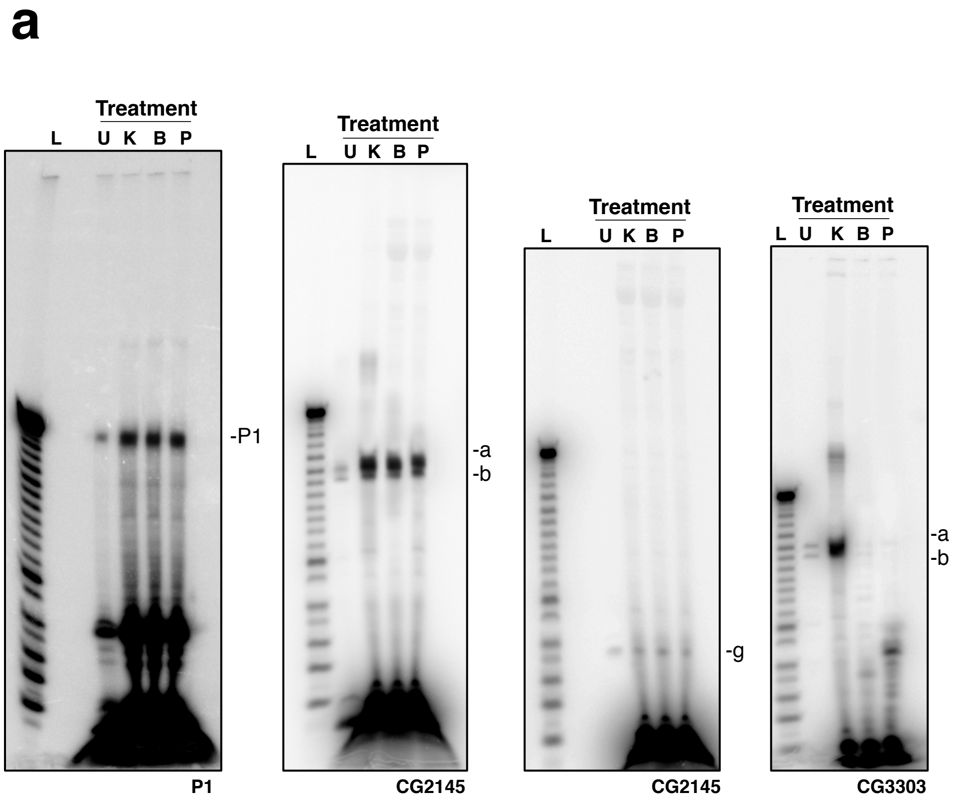

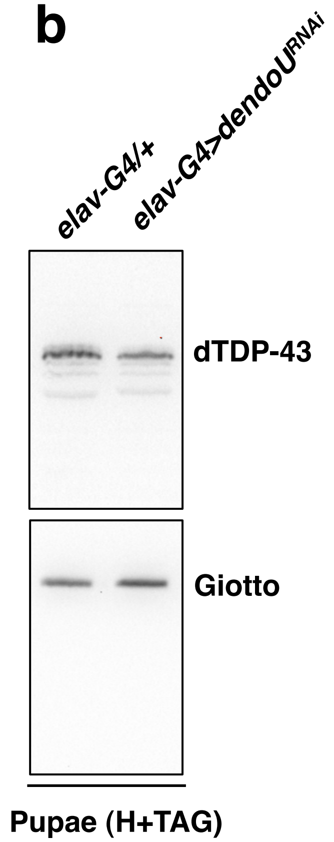

SUPPLEMENTARY VIDEO LEGENDS

Supplementary Video 1. CG2145 mutation does not impair fly behavioural performance and locomotor activity.
Related to Figure 4.
Movie showing the walking behaviour of CG2145 homozygous flies (genotype *w* P{EP}CG2145*^{G605}) during 30s on a plastic chamber, glass covered. Flies do not show locomotive defects.

Supplementary Video 2. Control flies do not exhibit alteration in locomotor activity.
Related to Figure 4.
Movie showing the walking behaviour of control flies (genotype *elav-G4/+*) during 30s on a plastic chamber, glass covered. Flies do not show locomotive defects.

Supplementary Video 3. Effects of *dendoU* inactivation on locomotor behaviour using the transgenic *dendoU*^{RNAi} line P{GD3933}v9916.
Related to Figure 4.
Movie showing the walking behaviour during 30s of *dendoU* interfered flies in post-mitotic neurons (*elav-G4>dendoU*^{RNAi}) (genotype *elav-G4/+; UAS-dendoU*^{RNAi}*/+*) during 30s on a plastic chamber, glass covered. Flies are severely uncoordinated and show dramatic locomotive defects.

Supplementary Video 4. Effects of *dendoU* inactivation on locomotor behaviour using an indipendent transgenic *dendoU*^{RNAi} line (P{KK115483}VIE-260B).
Related to Figure 4.
Movie showing the walking behaviour during 30s of *dendoU* interfered flies in postmitotic neurons (*elav-G4>dendoU*^{RNAi}) (genotype elav-*G4/+; UAS-dendoU*^{RNAi}*/+*) on a plastic chamber, glass covered. Flies are uncoordinated and show an impaired locomotor performance.

Supplementary Video 5. Effects of *dTDP-43* inactivation on locomotor behaviour.
Related to Figure 5.
Movie showing the walking behaviour of *dTDP-43* interfered flies in post-mitotic neurons (*elav-G4>dTDP-43*^{RNAi}) (genotype *elav-G4/+; UAS-dTDP-43*^{RNAi}*/+*) during 30s on a plastic chamber, glass covered. Flies show spastic and uncoordinated movements as previously described [Feiguin F. *et al.*, 2009]

SUPPLEMENTARY TABLES

Supplementary Table 1. Multiple sequence alignment of XendoU homologs retrieved from the NCBI nr database.
Related to Figure 1.
All sequences are indicated by the NCBI identifier. The sequences of the two *D. melanogaster* isoforms CG3303 and CG2145, human PP11 and *X. laevis* XendoU, shown in Figure 1, are bold and indicated by the UniProt identifier as well. Residues aligned to regions that are and are not present in the experimentally determined 3D structure of XendoU (Renzi et al., 2006) are upper- and lower-case, respectively. XendoU_SS: secondary structure elements detected in the 3D XendoU structure. Alpha-helices and beta-strands are indicated with α and β, respectively, and a number indicating their progression along the amino acid sequence. XendoU_FR: XendoU residues i) with a key functional role, i.e., those previously reported to reduce (c) or abolish (C) catalysis; affect RNA binding (R); in contact with the phosphate molecule in the XendoU 3D structure (P); or affecting both binding and catalysis (B); and ii) shown to interact with XendoU key-residues in the 3D structure (O). XendoU_# and XendoU_%: number and percentage of occurrence of each of the 20 residues or deletions at each alignment position. Positions corresponding to XendoU_FR or residues in α-helix and β-strand conformation have a green, yellow and cyan background, respectively. While XendoU "key" residues in the region 1-250 are conserved in >90% or >80% of the sequences, phosphate binding residues in the 251-292 region (i.e., N270, H272, G277, T278 and Y280) are conserved in ≤ 60% of the homologous sequences.

Supplementary Table 2. Summary of *dendoU* RNAi phenotypes in all neurons, cholinergic neurons, motor neurons and CCAP/bursicon neurons.
Related to Figures 4 and 5.

	Late pupal lethality (%)		immature phenotypes (%)		Lifespan		Performance index	
Neuronal driver	CTRL	*dendoU*^{RNAi}	CTRL	*dendoU*^{RNAi}	CTRL	*dendoU*^{RNAi}	CTRL	*dendoU*^{RNAi}
Pan-neuronal (elav^{C155})	ns	20	ns	100	32.07± 0.43d	2.05± 0.01d	nd	nd
Cholinergic neurons (Cha)	ns	ns	ns	100	31.06± 0.37d	7.57 ± 0.18d	8,38± 0.11	2,487 ± 0.12
Motor neurons (D42)	ns	ns	ns	10	31.38 ± 0.26d	21.53 ± 0.50d	7,75 ± 0.12	5,90 ± 0.11
CCAP/bursicon neurons	ns	ns	ns	ns	nd	nd	nd	nd

ns: not significant
nd: not done
